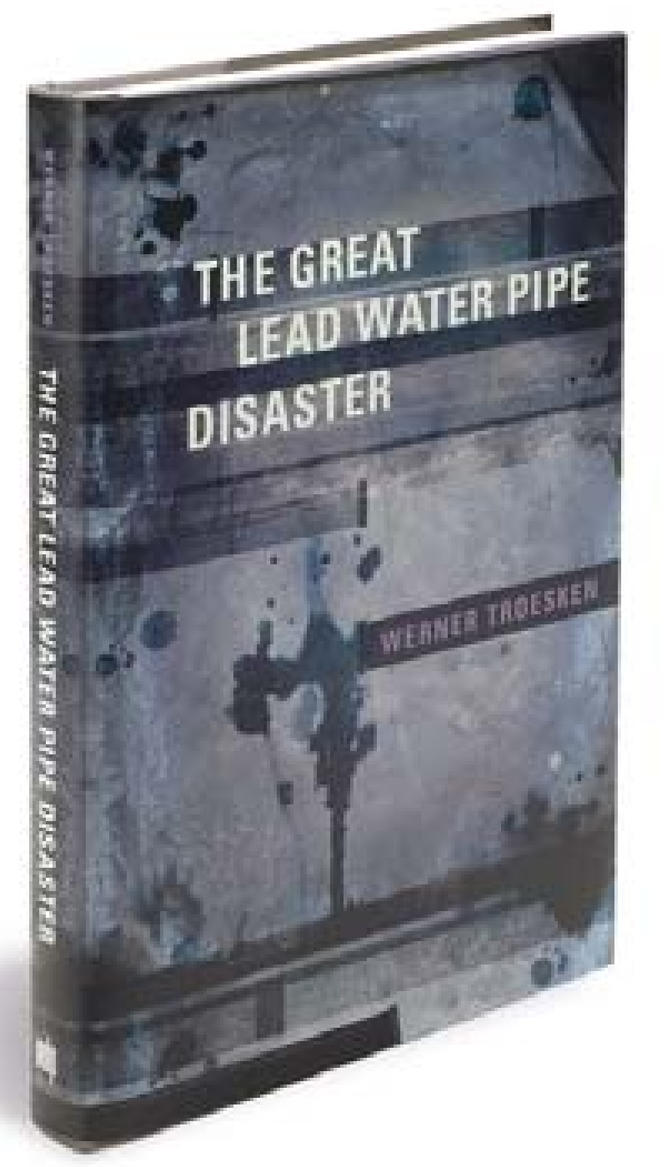# The Great Lead Water Pipe Disaster

**Published:** 2008-01

**Authors:** P. Barry Ryan

**Affiliations:** P. Barry Ryan is Professor of Exposure Assessment and Environmental Chemistry at Rollins School of Public Health of Emory University. His research focuses on understanding the mechanisms and factors influencing human exposure to environmental contaminants. He has served on numerous advisory boards and scientific panels. He currently is a member of the Board of Scientific Counselors for the U.S. Environmental Protection Agency Office of Research and Development

by Werner Troesken

Cambridge, MA:MIT Press, 2006. 318 pp. ISBN: 978-0-262-20167-4, $29.95

The adverse health outcomes associated with lead exposure were known in ancient times, but knowledge of adverse effects associated with high levels of exposure to lead and the realization that subtle (and not so subtle) effects may be occurring at much lower may not be readily seen by even the most astute scientists and physicians. This is the essential thesis of Werner Troesken’s *The Great Lead Water Pipe Disaster.*

In his well-researched and well-referenced text, Troesken develops his thesis on the effects of lead exposure in the context of the late 19th- and early 20th-century United States and United Kingdom. He begins with an anecdote on the mysterious death of Michael Galler, a resident of New York City, in 1868. The tale is filled with intrigue and even the suggestion that he might have been murdered by his wife. But eventually Troesken comes to implicate slow but insidious poisoning by lead leached from pipes delivering water to Galler’s home. Galler’s physician did not note any overt signs of “classic” lead poisoning, but exhumation and autopsy of Galler’s body revealed additional evidence of the ravages of long-term, low-level lead.

This prologue foreshadows the development of the book, as Troesken uses a combination of case studies of individual patients coupled with municipal water supply monitoring records and other scientific data to weave the tapestry of his thesis. Troesken’s story unfolds slowly, as did the development of understanding of the problem itself: It took nearly 50 years, from the 1880s through the early 1930s, before the medical and scientific community fully recognized the impact associated with low-level lead exposure. Initially, few believed that the levels of lead found in municipal water supplies could cause any harm at all. Although high exposures were known to cause effects, the observed low levels were deemed innocuous. Yet data showed higher fractions of spontaneous abortion, neurologic effects, and digestive problems in regions with lead water-delivery systems and acidic water supplies than in comparable regions without these factors. It took the development of epidemiology to uncover the associations.

Troesken offers compelling insight into the workings of both the medical and scientific communities and their interaction with the political structure of the time period. Although scientists and physicians were wrestling with the subtle and sometimes conflicting data on health outcomes associated with lead exposure, politicians, urban planners, and city leaders were wrestling with the costs, both fiscal and political, of assessing and fixing the problem. Readers may find similarities in this conflict between science and politics with current issues on climate change. As with climate change, early data collected on the effects of lead were uncertain and contradictory. However, eventually the scientific and medical communities came to a consensus that the lead pipe delivery systems were to blame, paralleling current thought on climate change data.

A professional historian, Troesken delves deeply into background material, seeking out primary references, private journals of physicians, and original data collected by numerous municipal water suppliers during this period. Although quite concerned with proper referencing and attribution, Troesken still makes the tale interesting, albeit somewhat tough sledding at points. The level of detail and the number of tables presented likely preclude this volume from reaching the best-seller list and may limit its accessibility to the lay reader. The end notes, which are likely to be skipped on a cursory reading, often add valuable insight and nuance to the story. The most challenging material, particularly the statistical analyses reflecting the impact of lead exposure on fetal mortality using regression methods, is relegated to one of three appendices totaling nearly 40 pages in length. In the end, however, environmental health scientists as well as those with keen interest in environmental health issues will doubtless find their efforts in getting through the material well rewarded.

Although an excellent read overall, the book is not without flaws. I was troubled by the mixing of units for lead concentration given in the text—grains per gallon, parts per million, and so on. Although in each case Troesken uses the unit appropriate for the era in which the data were collected, the constant switching back and forth was at times annoying and made interpretation more difficult. There are a few other minor flaws, but these are quibbles. On balance, Troesken’s book is quite solid and is recommended to all those interested in the history of this specific problem or of public health epidemiology in general.

## Figures and Tables

**Figure:**